# Evaluation of the Influence of Tantalum on the Microstructural, Mechanical and Electrochemical Properties of Ti–Mo–Zr–xTa Alloys for Use in Biomedical Devices

**DOI:** 10.3390/jfb17090434

**Published:** 2026-09-01

**Authors:** Cristina Jimenez-Marcos, Julia Claudia Mirza-Rosca, Madalina Simona Baltatu, Petricǎ Vizureanu

**Affiliations:** 1 Mechanical Engineering Department, Las Palmas de Gran Canaria University, 35017 Tafira, Spain; julia.mirza@ulpgc.es; 2 Materials Engineering and Welding Department, Transilvania University of Brasov, 500036 Brasov, Romania; 3 Department of Technologies and Equipments for Materials Processing, Faculty of Materials Science and Engineering, Gheorghe Asachi Technical University of Iasi, 700050 Iasi, Romania; madalina-simona.baltatu@academic.tuiasi.ro (M.S.B.); petrica.vizureanu@academic.tuiasi.ro (P.V.)

**Keywords:** vacuum arc remelting, titanium alloys, microstructure, microhardness, corrosion resistance

## Abstract

New titanium alloys for biomedical applications are being developed to avoid the use of aluminum and vanadium, which may raise concerns regarding their long-term biological effects. In this study, the effect of tantalum content on the microstructure, hardness and electrochemical behavior of Ti–Mo–Zr–xTa alloys (x = 5, 10 and 15 wt.%) obtained by vacuum arc melting (VAR) was investigated. Characterization included assessment of samples via optical microscopy, scanning electron microscopy (SEM) with energy-dispersive spectroscopy (EDS) analysis and X-ray diffraction (XRD). Mechanical response and corrosion resistance were assessed using Vickers microhardness and electrochemical tests in Ringer’s solution, respectively. The X-ray diffraction results indicated that the β phase was predominant in all three compositions. Furthermore, the increased tantalum content promotes the stability of this phase and reduces the α″ martensite contribution observed in alloys with lower tantalum content. The mean microhardness decreased as the tantalum content increased, although measurements showed less dispersion with increasing indentation load. All alloys exhibited passive behavior in Ringer’s solution, with the Ti–15Mo–7Zr–15Ta alloy showing the lowest corrosion current density and corrosion rate.

## 1. Introduction

The number of hip and knee arthroplasties performed in Europe and the United States has increased during the last decade, mainly because of population aging, obesity, and the growing prevalence of osteoarthritis [1,2]. However, the long-term durability of the implanted biomaterial is not guaranteed, as it can fail after 10 years of service life [3,4]. When a prosthesis fails, the patient needs to undergo a revision surgery, generally more complex, which involves a slower recovery for the patient, entails more risks and higher costs during the surgical procedure and therefore [3,4,5]. This situation demonstrates the difficulty of satisfactorily integrating mechanical, chemical, tribological and biological properties into a material to ensure its stable long-term performance [6,7].

In load-bearing metal implants, mechanical strength alone does not guarantee good clinical performance, as the material must also maintain its stability in the physiological environment, limit the release of metal species and transmit loads to the bone without significantly altering its natural remodeling [3,8,9]. Therefore, the problem is not only chemical or biological, but also mechanical: the distribution of loads between the implant and the surrounding bone affects both the tissue response and the durability of the device [8,10,11,12]. These requirements have placed titanium and its alloys among the most commonly used materials in orthopedic, dental and cardiovascular applications, due to their low density, specific strength, corrosion resistance and generally favorable biological response [13,14,15,16].

Commercially pure titanium (cp–Ti) and grade 5 alloy Ti–6Al–4V remain benchmark materials in the design process of metallic implants [17,18,19,20]. Ti–6Al–4V is widely used in dental devices, joint prostheses, and bone fixation components because of its mechanical strength, fatigue resistance and corrosion resistance in physiological environments [16,21]. Nevertheless, aluminum (Al) ions are linked to neurodegenerative diseases (e.g., Alzheimer’s) and vanadium (V) ions are cytotoxic and carcinogenic. Furthermore, its elastic modulus (~110 GPa) is significantly higher than that of cortical bone (~10–30 GPa), leading to stress-shielding and bone resorption [21,22,23]. Ni–Ti (Nitinol) contains nickel (Ni) which is a well-known allergen and can cause severe allergic reactions in sensitive individuals. There are also concerns about the toxicity and carcinogenic potential of nickel ions released over time [17,24]. Although cp–Ti is biocompatible, it lacks the mechanical strength and wear resistance needed for high load-bearing applications like hip and knee implants. Its elastic modulus is lower than that of Ti–6Al–4V but remains higher than that of cortical bone.

These limitations have promoted the development of β-type titanium alloys free of Al, V, and Ni. The β phase has a body-centered cubic structure [10,12], which allows for lower elastic moduli compared to those of conventional α+β alloys [22,23,25]. To stabilize this phase, elements such as niobium (Nb) [26,27], molybdenum (Mo) [21,25,28], tantalum (Ta) [29,30], zirconium (Zr) [26,31,32], iron (Fe) [33,34] or silicon (Si) [35] are used, selected for their effect on the microstructure, corrosion resistance, hardness and biological response.

The Ti–Nb–Ta–Zr systems have shown low elastic moduli and favorable biological behavior [36,37]. The microstructure, hardness and resistance to corrosion of Ti–Nb–Zr–Si [38], Ti–Nb–Zr–Fe [39] and Ti–Mo–Zr–Ta [40,41] were also evaluated to determine how stabilizing and hardening elements impacted these properties.

Among the alloying elements used in β-type titanium alloys, Mo, Zr and Ta are of particular interest. Mo is a strong β-stabilizing element and can increase the mechanical strength of titanium alloys. Zr is generally considered a neutral element with good metallurgical compatibility with Ti and may contribute to corrosion resistance and microstructural refinement. Ta also promotes β-phase stability and exhibits favorable biological behavior. In addition, the high stability of Ta_2_O_5_ may improve the protective properties of the passive film formed in physiological environments. Therefore, the combined addition of Mo, Zr and Ta offers the possibility of adjusting the phase constitution, mechanical response, and corrosion behavior of titanium alloys [42].

Even so, there are still few studies that systematically analyze how increasing Ta content modifies, in Ti–Mo–Zr alloys, the microstructure, microhardness and electrochemical response in Ringer’s solution.

The production of Ti–Mo–Zr-–Ta alloys is metallurgically complex because Mo and Ta are refractory elements with high melting temperatures. Their incorporation requires appropriate melting conditions and atmospheric control to limit oxidation and promote chemical homogeneity. Therefore, in this work, vacuum arc remelting (VAR) is employed as the manufacturing route. This technique allows the constituent elements to be melted under a controlled atmosphere and permits repeated melting cycles to improve their redistribution [43]. Nevertheless, because of the substantial differences in melting point, density, and solidification behavior among Ti, Mo, Zr, and Ta, local microsegregation may still develop, particularly between dendritic and interdendritic regions.

Electrochemical testing in Ringer’s solution allows the corrosion behavior of the alloys to be compared in an electrolyte containing ions commonly present in physiological fluids. Although the tests are conducted at room temperature, the use of the same controlled conditions allowed the passive behavior of the different compositions to be compared. Moreover, this technique allows obtaining parameters such as open-circuit potential, corrosion current density and stability of the passive surface film. In Ti–Mo–Zr-–Ta alloys, the passive film may contain oxides associated with the constituent elements, so its stability will depend on both the chemical composition and the microstructure generated during solidification.

In this research, Ti–Mo–Zr–xTa alloys with Ta contents of 5, 10 and 15 wt.% were fabricated and characterized using the VAR process. The influence of Ta content on their microstructure, phase constitution, Vickers microhardness and electrochemical behavior in Ringer’s solution at 25 °C was evaluated. The results provide information that enables the creation of new Al- and V-free titanium alloys with good potential for use in new biomedical devices that exhibit a higher degree of electrochemical stability and improved surface characteristics.

## 2. Materials and Methods

### 2.1. Alloy Production and Specimen Preparation

A vacuum arc remelting (VAR) furnace MRF ABJ 900 (Allenstown, NH, USA) was used to manufactured the following alloys from pure alloying elements (Ti, Mo, Zr and Ta): VAR1 (73% Ti, 15% Mo, 7% Zr, 5% Ta), VAR2 (68% Ti, 15% Mo, 7% Zr, 10% Ta) and VAR3 (63% Ti, 15% Mo, 7% Zr, 15% Ta). Melting was performed under a high-purity argon atmosphere and each alloy was remelted six times, with three melting cycles performed on each side, to improve the distribution of the alloying elements.

**Figure 1:**
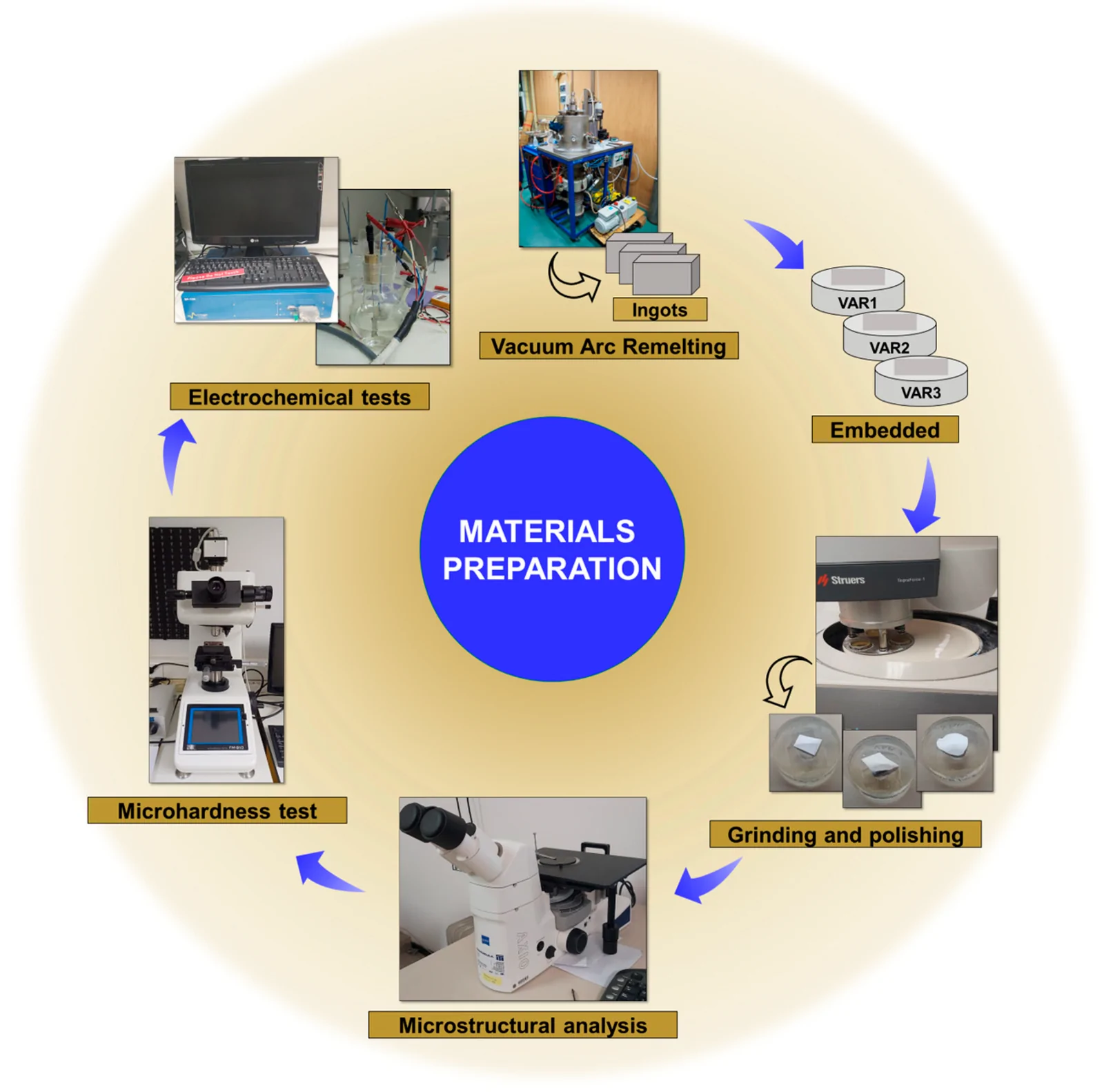
Production and metallographic preparation of the Ti15Mo7ZrxTa alloy specimens.

The methodology previously verified in comparable research [44,45] was followed for sample preparation. Prior to microstructural, mechanical, and electrochemical characterization, the specimens were cut, mounted, ground and polished using typical metallographic techniques until a mirror-like surface smoothness was achieved (see Figure 1).

### 2.2. Microstructural Characterization

Metallography, scanning electron microscopy (SEM) with energy-dispersive X-ray spectroscopy (EDS) and X-ray diffraction (XRD) were used to examine the microstructure and phase constitution of the samples.

A Zeiss Axio Imager A1 (Oberkochen, Germany) optical microscope was used to take micrographs of the materials’ surfaces at various magnifications in order to study their microstructure. First, each sample was submerged in a chemical reagent (10 mL HF, 5 mL HNO_3_ and 85 mL H_2_O) for 30 s.

The microstructure analysis was conducted at an accelerating voltage of 20 kV using the scanning electron microscope (SEM) Tescan Vega LMH II (Brno, Czech Republic) fitted with an energy-dispersive X-ray spectroscopy (EDS) Bruker detector.

The materials were subjected to phase analysis using a PANalytical X’Pert Pro MPD diffractometer (Almelo, The Netherlands). The research was conducted using a CuK radiation (1.54051 Å) in the range of 2θ = 10–90°, with a step size of 0.13°, time/step of 51 s.

### 2.3. Microhardness Measurements

In accordance with ISO 6507-1:2018 [46], three independent specimens of each alloy condition (VAR1, VAR2, and VAR3) were tested using a Future Tech FM-810 hardness tester (Kawasaki, Japan). Twelve measurements were obtained for each load applied to each specimen (in this case, 5, 25 and 50 gf), with each measurement lasting 15 s. For each load, the twelve indentations were performed along a single linear path across the polished surface of each specimen, resulting in three separate and approximately parallel paths, one for each applied load. A minimum center-to-center distance of 500 µm was maintained between adjacent indentations, while the distance from the center of the indentations to the specimen edge was also maintained at a minimum of 500 µm. Thus, a total of 36 indentations were performed on each specimen, corresponding to 108 measurements for each alloy condition. The computer then automatically computed the Vickers microhardness values using the measured diagonal lengths.

### 2.4. Electrochemical Tests

In order to ascertain the corrosion behavior of the materials under investigation, the three samples (working electrodes) were immersed in an electrochemical cell containing a Grifols Ringer Lactate (Barcelona, Spain) electrolyte at 25 °C, with a saturated calomel electrode (SCE) and a platinum (Pt) electrode. The solution was used directly from the commercial container without prior deaeration. After the samples were submerged, the potentiostat-galvanostat BioLogic Essential SP-150 (Seyssinet-Pariset, France) was employed to measure the open circuit potential over time using OCP vs. Time, obtain corrosion rate (CR) values using linear polarization (LP) and determine the samples’ impedance values using electrochemical impedance spectroscopy (EIS).

#### 2.4.1. Open Circuit Potential

During 24 hours of immersion in Ringer solution, the open-circuit potential (OCP) of each sample was evaluated using the EC-Lab V.9.55 program’s OCP vs. time approach. The corrosion potential was continuously monitored, and data were recorded at 300 s intervals or whenever the potential shifted by more than 200 mV.

#### 2.4.2. Potentiodynamic Polarization

The potentiodynamic polarization tests were carried out using the “LP” technique. The potential was scanned from −1.0 to 2.0 V vs. SCE at a scan rate of 20 mV/min (0.33 mV/s) and the data were collected at 0.5 s intervals. The “Tafel Fit” approach was used to determine the corrosion rate (CR) of each sample and the potentiodynamic polarization curves were plotted, following ASTM G102-23 [47]. The linear regions of the anodic and cathodic branches adjacent to the corrosion potential were selected for the Tafel extrapolation.

#### 2.4.3. Electrochemical Impedance Spectroscopy (EIS)

In accordance with ISO 16773-1:2016 [48], impedance spectra were acquired in the region of ± 300 mV vs. SCE in order to characterize the oxide layer using EIS. At each potential, the system was allowed to equilibrate for five minutes. The frequency ranges of 200 kHz to 100 mHz was scanned. An electrical equivalent circuit (EEC) was used to fit the experimental data and Nyquist and Bode diagrams were utilized for presenting the results. The quality of the fitting was evaluated using the chi-squared value.

## 3. Results and Discussion

While many studies explore binary Ti–Ta alloys [29,49], the current work investigates the complex synergistic effect of Mo and Zr in addition to Ta. This is an innovation because it allows for more sophisticated tailoring of properties. Mo acts as a strong β-stabilizer and strengthens the alloy, while Zr contributes to its biocompatibility and corrosion resistance. The study’s value lies in understanding how these elements interact with Ta to influence the final properties, which is not addressed by simpler binary systems. Studies like [26] emphasize the importance of wear resistance for joint prostheses. The current work does not analyze how the microstructure and hardness (which decreased with Ta) would affect the wear and tribocorrosion performance. Another study [18] highlights that high corrosion resistance does not always guarantee good fatigue resistance in a corrosive environment. This is a critical property for load-bearing implants that is not addressed. However, most published studies have focused on binary Ti–Ta alloys or multicomponent systems in which several alloying elements were simultaneously modified, making it difficult to isolate the specific contribution of Ta. In contrast, the present study maintains constant Mo and Zr concentrations while varying only the Ta content, allowing a direct evaluation of the relationship between Ta addition, phase constitution, hardness and electrochemical behavior.

### 3.1. Microstructural Characterization

**Figure 2:**
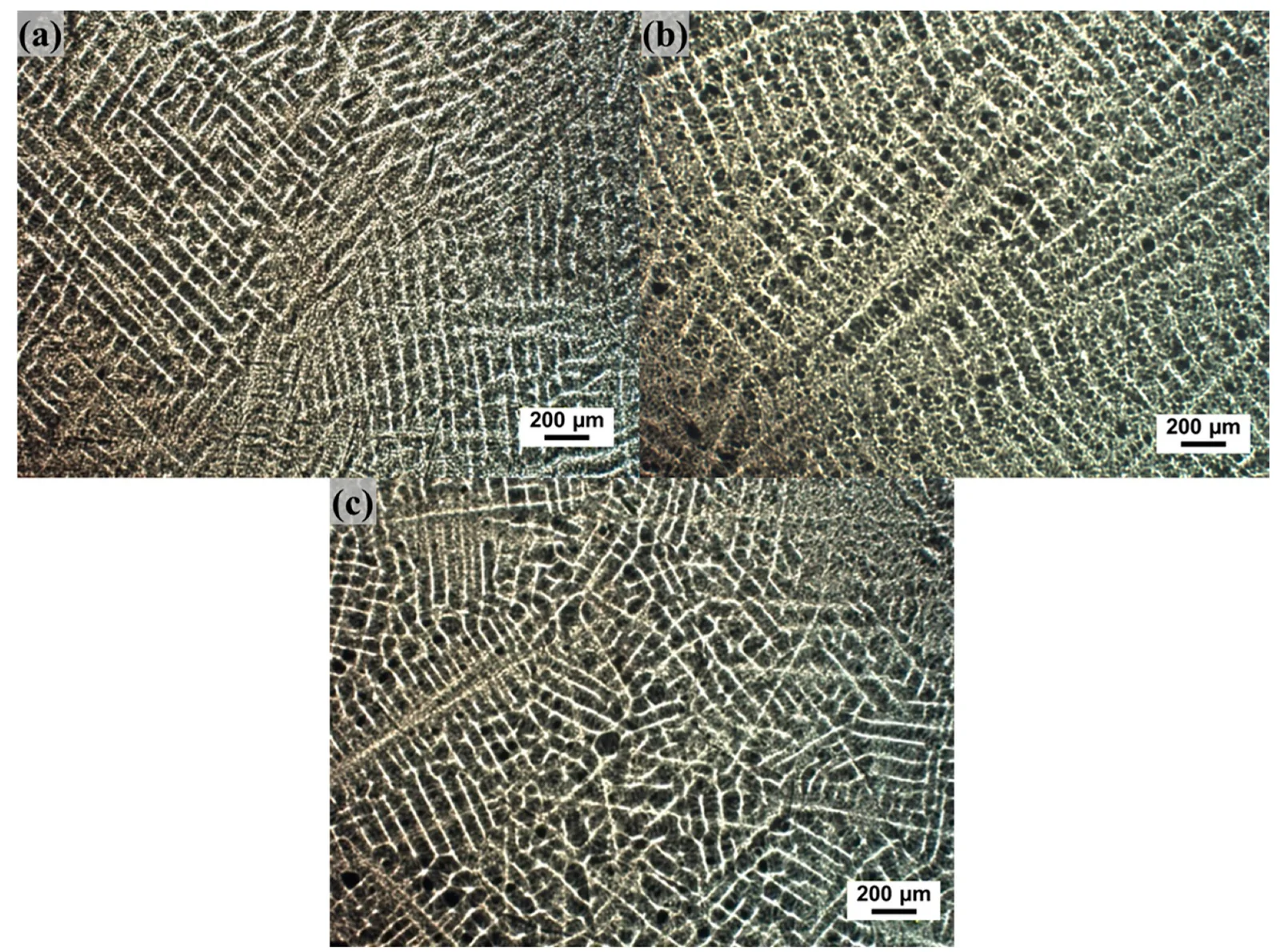
Optical microstructures for (**a**) VAR1, (**b**) VAR2 and (**c**) VAR3 samples after chemical etching.

The optical micrographs of VAR1, VAR2 and VAR3 samples following chemical etching are exhibited in Figure 2. In all three samples, a dendritic microstructure characteristic of the VAR solidification process is observed.

For VAR1, containing 5 wt.% Ta, the dendritic structure is clearly visible, with continuous dendrite arms and a noticeable contrast between dendritic and interdendritic areas. As the Ta content increases to 10 wt.% (VAR2), it appears comparatively coarser and more heterogeneous, with local variations in the size and orientation of the dendritic features. For 15 wt.% Ta (VAR3), the dendritic structure is clearly observed again, with a tighter network and a more compact pattern.

Overall, these observations suggest that the Ta content influences the morphology and etching response of the solidified microstructure.

**Figure 3:**
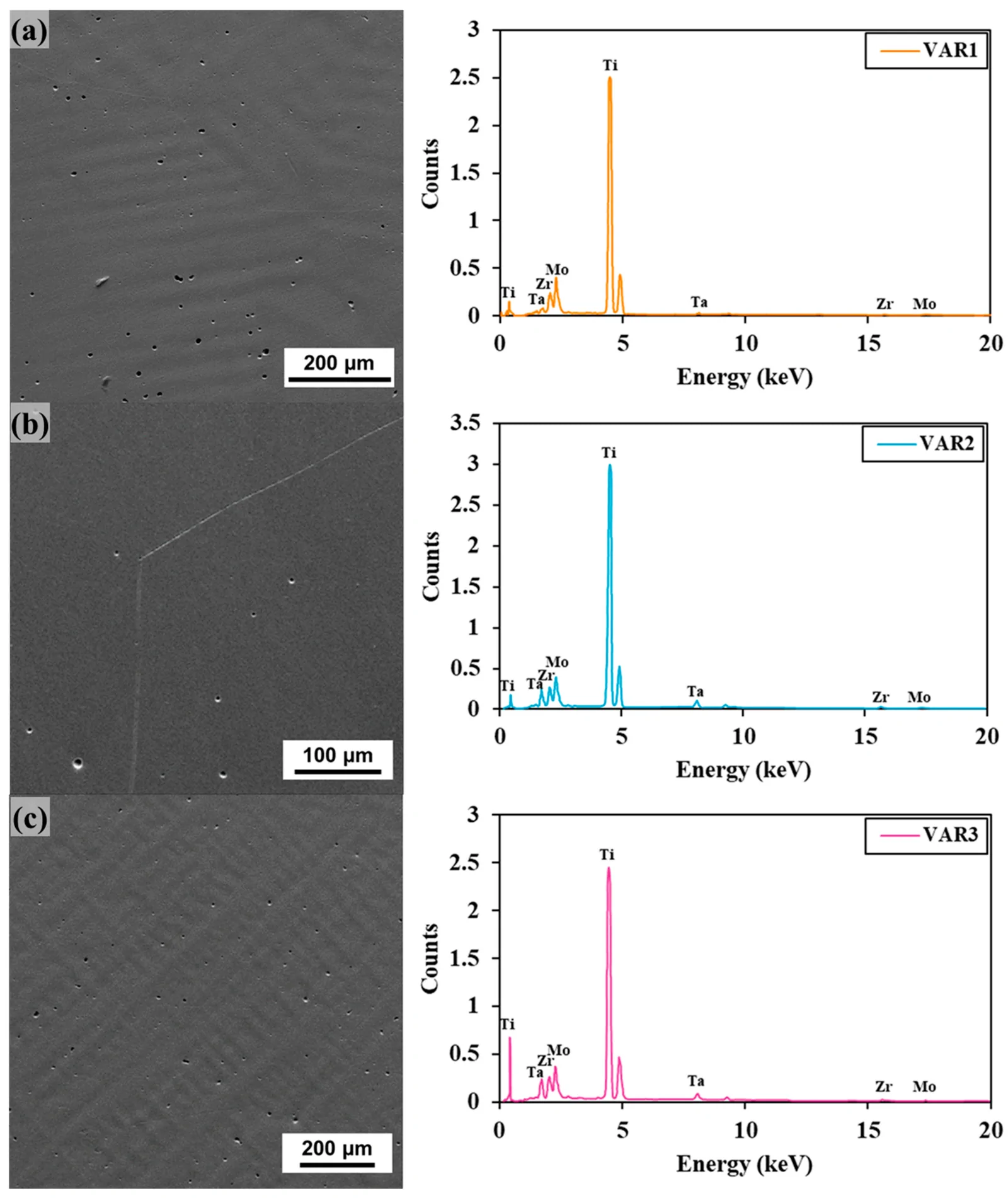
SEM surface morphologies and EDS spectra for VAR1 (**a**), VAR2 (**b**) and VAR3 (**c**). The spectra are independently scaled for visualization and are intended for qualitative elemental identification; therefore, peak heights should not be used for direct quantitative comparison between the samples.

Figure 3 provides SEM images and the corresponding EDS spectra for the VAR1, VAR2 and VAR3 alloys. All samples exhibit a generally continuous surface containing isolated pores, without large defects or evident discontinuities at the investigated scale. The pore volume fraction was not quantitatively determined in the present study. The observed porosity may locally influence the microhardness values when an indentation is positioned near a pore and may also promote localized electrolyte accumulation, thereby affecting the corrosion response. In this sense, the specific contribution of porosity to the measured mechanical and electrochemical properties cannot be separated from the effects of alloy composition and microstructure and represents a limitation of the present study.

**Figure 4:**
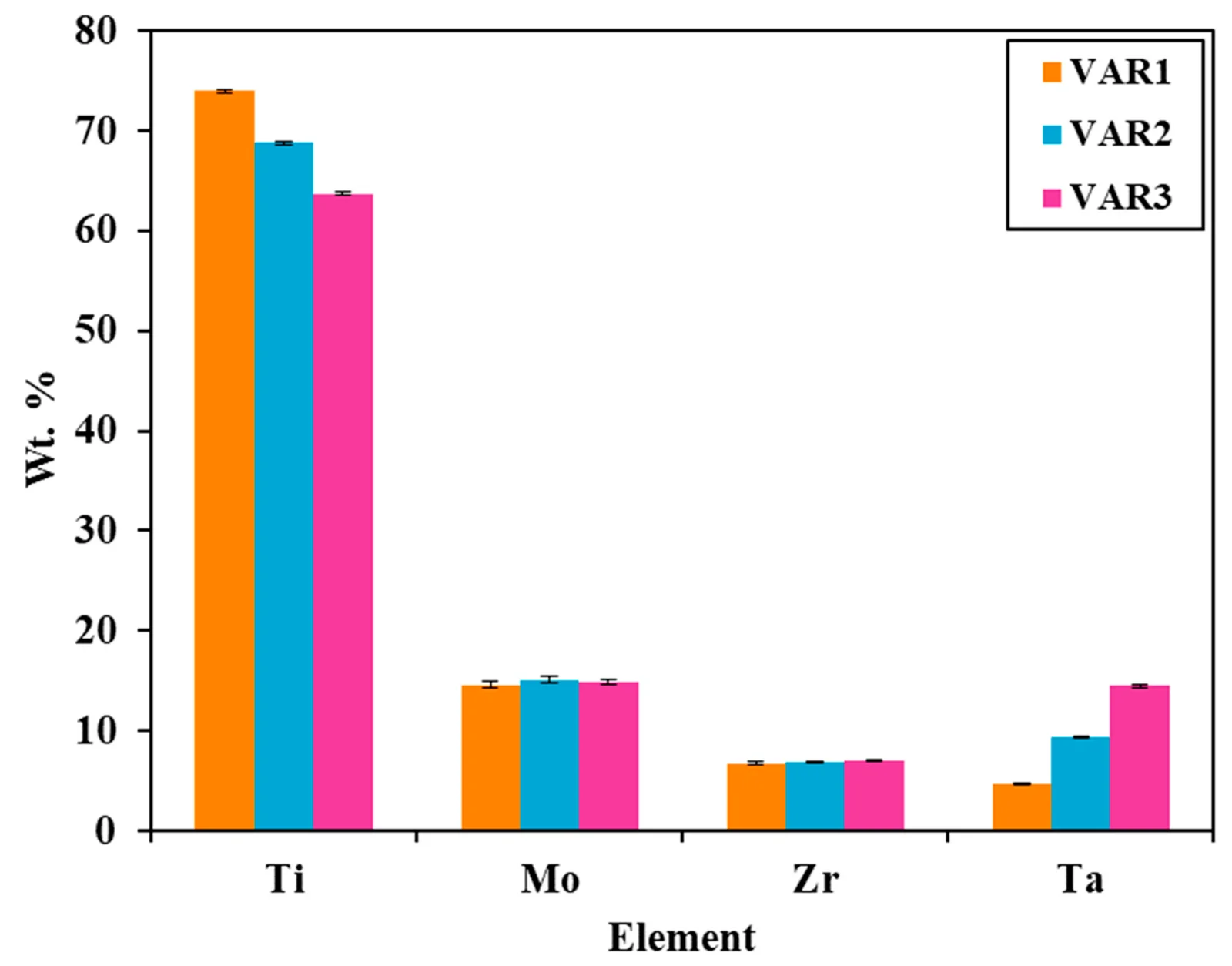
Quantitative EDS results for the VAR1, VAR2 and VAR3 alloys, expressed in wt.%.

The presence of the primary alloying elements (Ti, Mo, Zr and Ta) in the three samples is confirmed by the EDS spectra (see Figure 3). In all three spectra, the Ti peaks show the highest intensity, as expected from its role as the main element of the alloy. The peaks corresponding to Mo and Zr appear with lower intensity, while the Ta signal progressively increases from VAR1 to VAR3, in line with the alloys’ increasing Ta concentration. The quantitative EDS results are presented in Figure 4.

**Figure 5:**
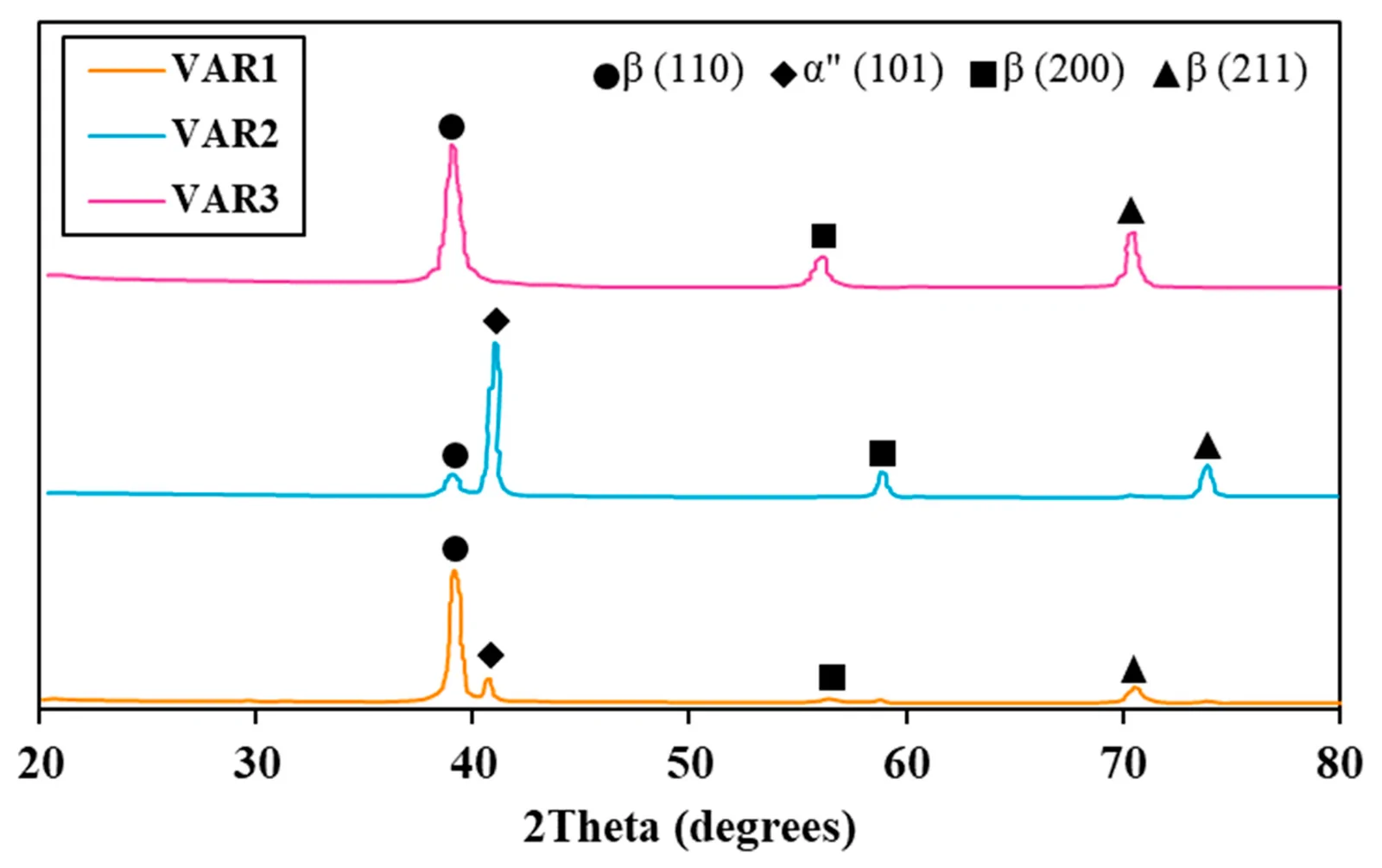
X-ray diffraction patterns of the for VAR1, VAR2 and VAR3 samples.

The X-ray diffraction patterns of the VAR1, VAR2 and VAR3 samples are presented in Figure 5. In all three samples, peaks associated with the β phase are primarily identified, confirming the β-stabilized nature of the Ti–Mo–Zr–Ta system. This phase appears with greater intensity around 40°, along with secondary reflections near 56–58° and 70–74°.

In VAR1 and VAR2, a signal attributed to phase α″ is also observed, located around 38–40°. Its presence indicates that, in compositions with lower Ta content, part of the β phase can transform into orthorhombic α″ martensite. In contrast, in VAR3 the pattern is dominated by the β phase, suggesting a greater stabilization of the β phase with an increase in Ta content.

Figure 5 shows small differences in the positions of the α″ martensite and β-phase reflections between VAR1 and VAR2. These differences may be associated with the variation in Ta accommodated in the Ti-based phases, which can produce composition dependent lattice strain and changes in the corresponding interplanar spacings. According to Bragg’s law, shifts towards higher 2θ values indicate lower interplanar spacings, whereas shifts towards lower 2θ values indicate higher interplanar spacings. However, the reflections assigned to α″ martensite and the β phase in the region near 40° are closely positioned and may partially overlap. Therefore, the apparent displacement of the diffraction maximum may also be influenced by changes in the relative contributions of the two phases, these peak shifts are interpreted qualitatively as being consistent with Ta-related lattice distortion.

### 3.2. Vickers Microhardness

**Figure 6:**
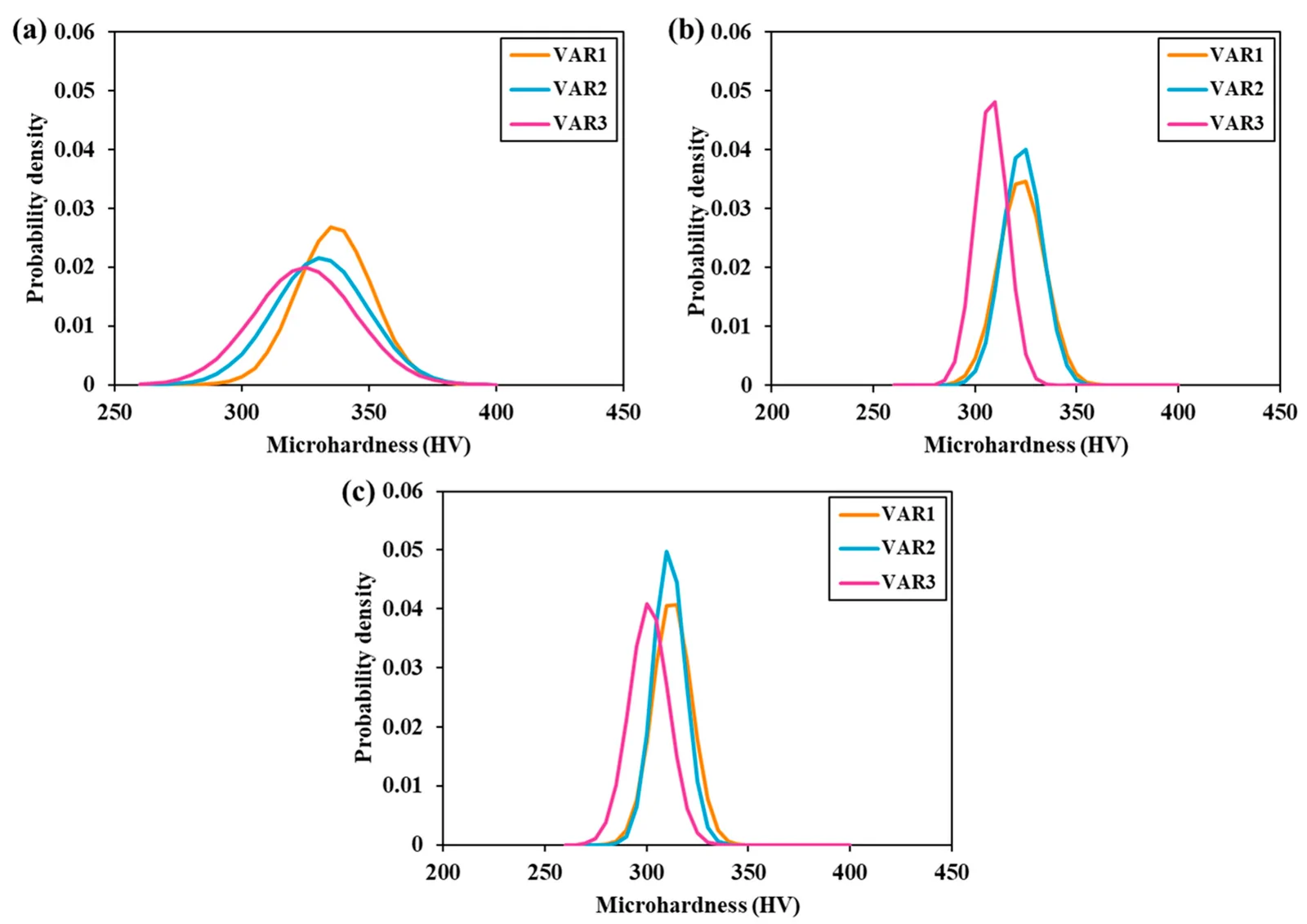
Normal distributions fitted to the Vickers microhardness values measured along linear indentation paths across the polished surfaces of the VAR1, VAR2 and VAR3 specimens at applied loads of (**a**) 5 gf, (**b**) 25 gf and (**c**) 50 gf.

Statistical microhardness values are presented in Table 1, including the mean, median, maximum and minimum values, standard deviation (SD), and the coefficient of variation (CV). The measurements were obtained from indentations arranged linearly across the polished surface of each specimen. Likewise, Figure 6 shows the Vickers microhardness distributions for the VAR1 VAR2 and VAR3 alloys obtained using loads of 5, 25 and 50 gf. For each specimen, the indentations corresponding to the three applied loads were arranged along three separate linear paths across the polished surface.

At 5 gf, the hardness values are quite scattered for the three alloys, and this is also reflected in the coefficients of variation. The distributions show a considerable overlap, with mean hardness values of 336 HV, 331 HV and 325 HV for VAR1, VAR2 and VAR3, respectively. VAR3 shows the widest dispersion, followed by VAR2, whereas VAR1 gives a more consistent response. This behavior is expected at such a low load, since the indentation is more affected by local features of the surface and by small microstructural differences.

When the load is increased to 25 gf, the curves become narrower and the coefficients of variation decrease in all cases. VAR1 and VAR2 show the same mean hardness value (323 HV), while VAR3 decreases to 308 HV, making the differences between the alloys more evident. This suggests that the effect of local heterogeneities becomes less important as the indentation area increases.

At 50 gf, the measurements are more stable and the coefficients of variation reach their lowest values, especially for VAR2. The same order is maintained, with VAR1 showing the highest mean hardness (313 HV), followed by VAR2 (311 HV) and VAR3 (301 HV). The narrower distributions indicate improved repeatability of the measurements, while the increase in indentation depth with increasing load is accompanied by a slight decrease in hardness values.

Ta is a β-stabilizing element and when added to the Ti–Mo–Zr alloy system, it dissolves into the β-phase matrix. As indicated by the Mo equivalent concept, an increase in Ta content raises the overall β-stabilizing effect and this suppresses the transformation of the high-temperature β-phase to the orthorhombic α″ martensite upon cooling. The XRD results show that VAR1 (5% Ta) and VAR2 (10% Ta) contain a mixture of β and α″ phases, while VAR3 (15% Ta) is predominantly β. The α″ martensite is a harder phase than the β-phase. Therefore, as Ta increases and the fraction of the softer β-phase increases (while α″ decreases), the overall microhardness of the alloy decreases. This is consistent with the experimental data (VAR1 showing the highest hardness and VAR3 the lowest).

### 3.3. Electrochemical Tests

#### 3.3.1. E_corr_ vs. Time (OCP)

**Figure 7:**
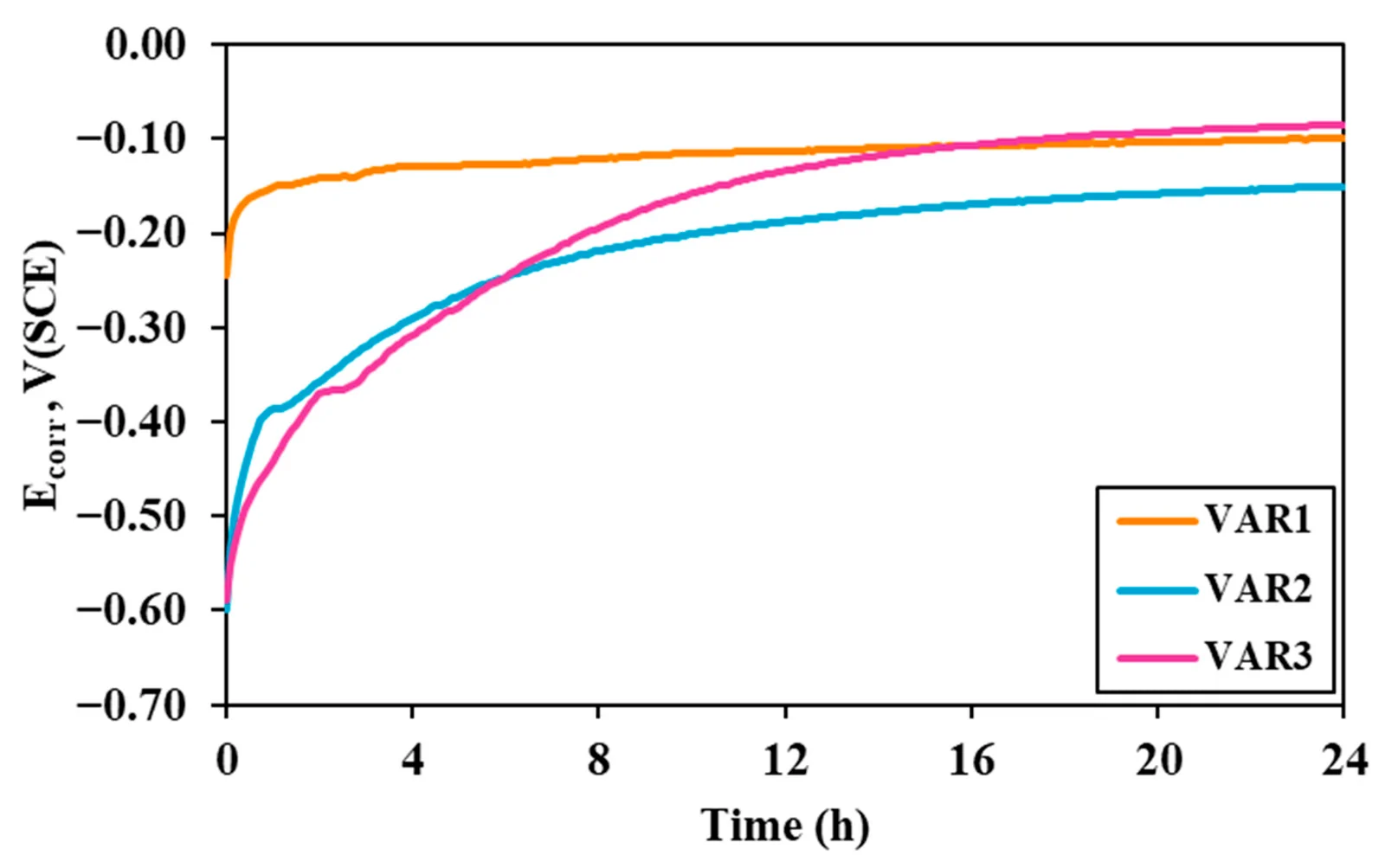
Open circuit potential evolution of the VAR1, VAR2, and VAR3 alloys during 24 h of immersion in Ringer lactate solution.

Figure 7 presents the evolution of the open circuit potential of VAR1, VAR2 and VAR3 during 24 h of immersion in Ringer’s solution. At the beginning of the test, VAR2 and VAR3 show very similar potential values, close to −0.60 and 0.59 V versus SCE, respectively, whereas VAR1 starts from a less negative value of −0.24 V. During the first 4 h, the three alloys shift towards less negative potentials, although this change is more noticeable for VAR2 and VAR3, which reach approximately −0.29 and −0.31 V, compared with −0.13 V for VAR1. Between 4 and 16 h, VAR2 and VAR3 continue to move slowly in the positive direction, while VAR1 remains almost unchanged. Around this time, VAR1 and VAR3 reach similar E_corr_ values, close to −0.11 V, suggesting the gradual development of a surface layer that limits metal dissolution. After 16 h, the three curves tend to stabilize and only small changes are observed up to the end of the test. After 24 h, VAR1 and VAR3 show the least negative potentials, around −0.09 and −0.10 V, while VAR2 remains slightly lower, near −0.15 V.

Overall, VAR1 has shown greater stability since the start of the test. VAR2 and VAR3 require more time to reach a similar state, particularly VAR3, which exhibits the most pronounced change during the first few hours. The trend toward less negative values in all three cases is consistent with the formation of a surface film that limits electrochemical activity, although its effect is not the same in all alloys.

#### 3.3.2. Potentiodynamic Polarization

**Figure 8:**
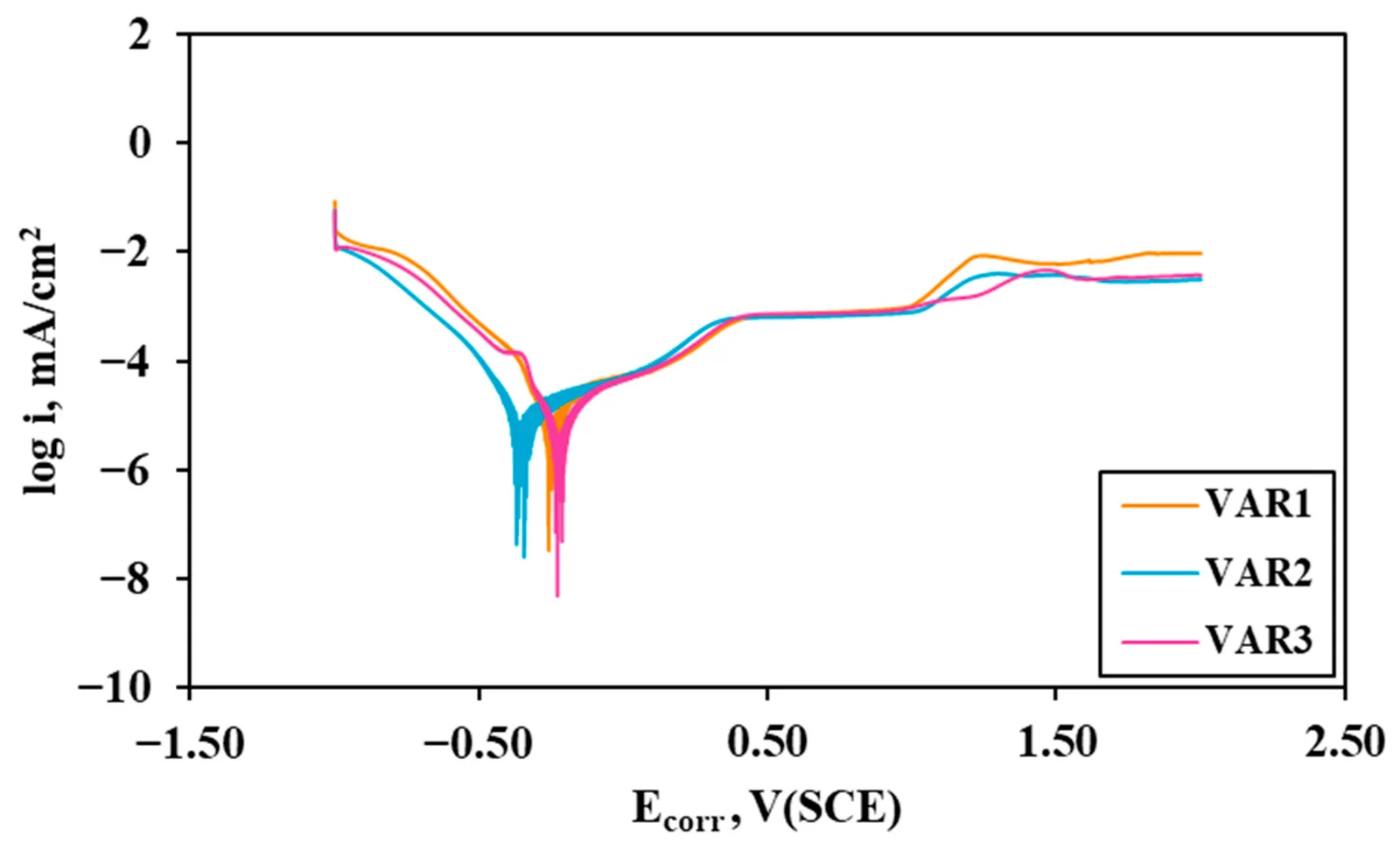
Potentiodynamic polarization curves of the VAR1, VAR2, and VAR3 alloys in Ringer lactate solution at 25 °C.

Figure 8 presents the potentiodynamic polarization curves of the VAR1, VAR2, and VAR3 alloys, while Table 2 summarizes the specimen parameters and the electrochemical values obtained from the Tafel analysis. In the potentiodynamic plot, the cathodic (left) and anodic (right) branches are clearly distinguishable on either side of the corrosion potential.

Following the Tafel Fit adjustment, in the analyzed range from −1 V to 2 V, the sample with the best combination of E_corr_ and current density (i_corr_) parameters is VAR3, followed by VAR1 and then VAR2. This reduction indicates a lower dissolution rate in the alloy with the higher Ta content.

From the extrapolation point of E_corr_ and i_corr_ toward more positive potentials, the three alloys exhibit anodic behavior, with no distinct passivation region observed in the analyzed range.

Differences between materials are primarily evident in the current levels reached in the anode branch. VAR3 exhibits lower current densities than VAR1 and VAR2, which is consistent with its lower i_corr_ value and slower corrosion rate. VAR1 and VAR2 show similar polarization responses and higher current densities over most of the investigated anodic range.

Tafel slopes also reflect changes in the electrochemical response. VAR3 has a lower anodic slope (β_a_) than the others, suggesting different behavior in the anodic branch. VAR2, on the other hand, exhibits the highest anodic slope. In the cathodic branch, the differences are smaller, although VAR1 exhibits a slightly higher cathodic slope (β_c_).

Also, it can be seen that the calculated corrosion rates (CRs) were much lower for VAR3 (4.82 × 10^−6^ mm/year) than for VAR1 and VAR2 (1.05 × 10^−4^ mm/year). It should be mentioned that in comparable environments, the CR findings were lower than those of commercial materials like Ti6Al4V (1.07 × 10^−4^ mm/year) and CP–Ti (4 × 10^−4^ mm/year) [50,51]. The effect of Ta becomes more evident when its content is higher, probably due to its influence on the stability of the surface film formed during the test.

#### 3.3.3. Electrochemical Impedance Spectroscopy (EIS)

**Figure 9:**
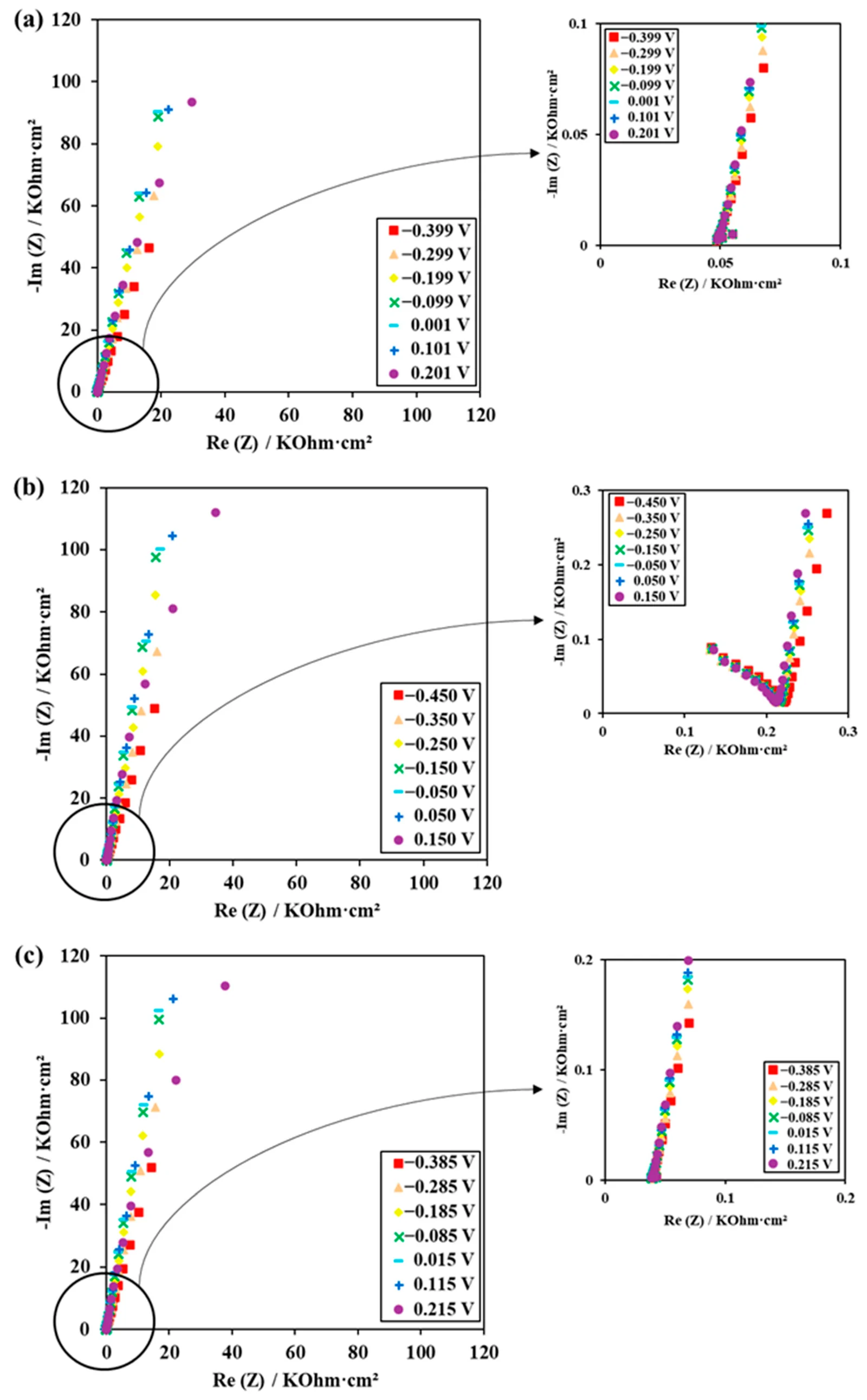
Nyquist diagrams of experimental data with magnified detail of the boxed area for (**a**) VAR1, (**b**) VAR2 and (**c**) VAR3.

Figure 9 shows the Nyquist diagrams for the alloys VAR1, VAR2 and VAR3 obtained at ±300 mV versus SCE. The formation of wider arcs is linked to greater corrosion resistance, while the deviation from a perfect semicircle suggests non-ideal capacitive behavior, possibly related to surface inhomogeneity or diffusion effects.

Throughout the potential scan applied to the SCE, both the real and imaginary components of the impedance tend to increase as the applied E_corr_ becomes less negative for all three samples. The relative impedance response of the three alloys depends on the applied potential, and no single alloy exhibits the highest resistance at every potential. Nevertheless, VAR3 shows comparatively high impedance values at several potentials and also exhibits the lowest corrosion current density in the polarization measurements. These results suggest that the increase in Ta content may influence the electrochemical response of the surface film, although its chemical composition was not determined directly.

**Figure 10:**
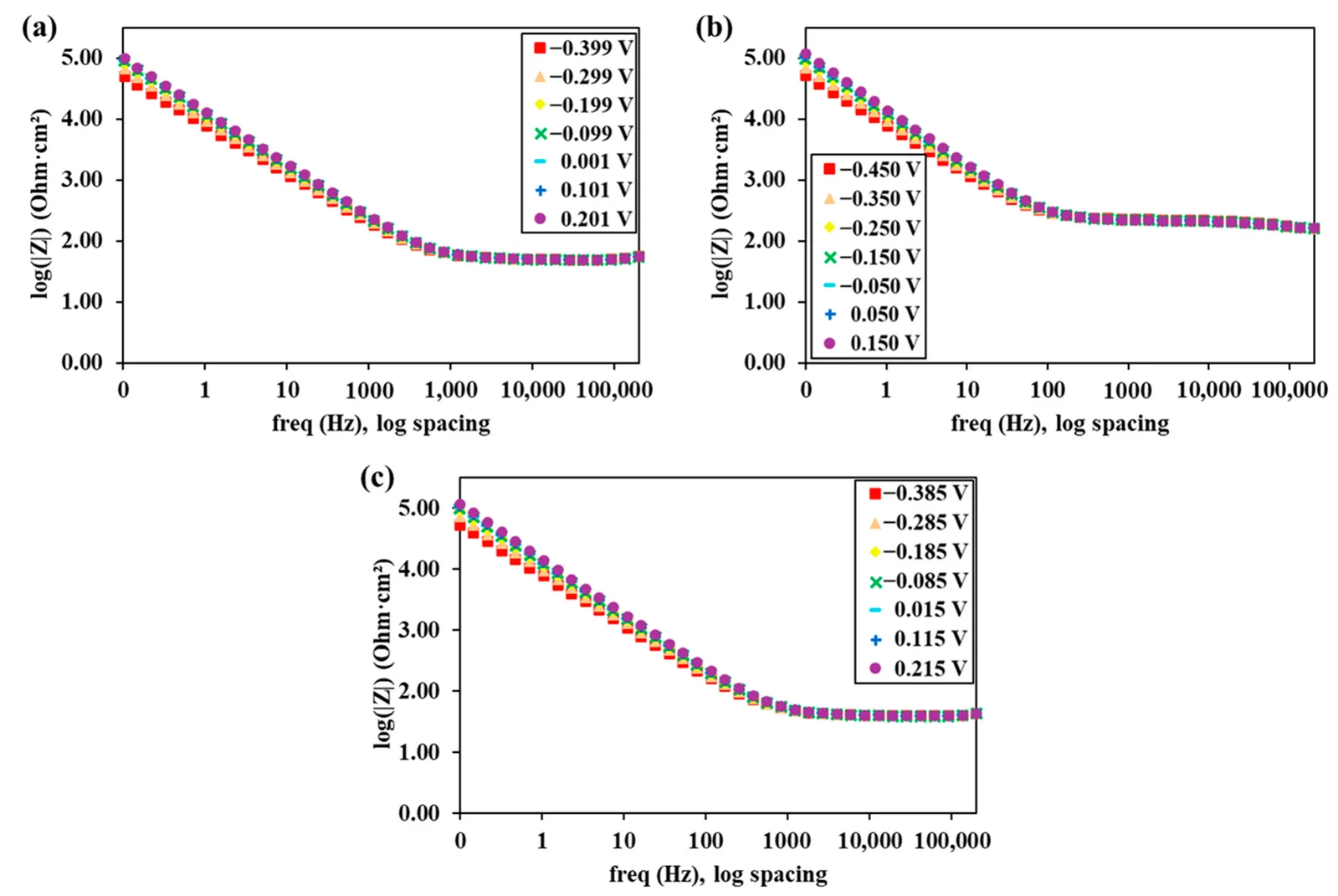
Bode-|Z| diagrams of experimental data for (**a**) VAR1, (**b**) VAR2 and (**c**) VAR3.

Figure 10 displays the Bode impedance modulus graphs of the samples submerged in Ringer’s solution, obtained at ±300 mV relative to the SCE. At high frequencies, it is observed that the values of |Z| are low and increase as the frequency value decreases. At this initial point, it is observed that the curves of the different potentials of each sample exhibit similar behavior and from 100 Hz onward, it can be seen that the values of |Z| increase when applying more positive potentials. Similar to the Nyquist diagram, the maximum impedance values were obtained by VAR2 (117.26 KOhm·cm^2^) and VAR3 (116.61 KOhm·cm^2^).

**Figure 11:**
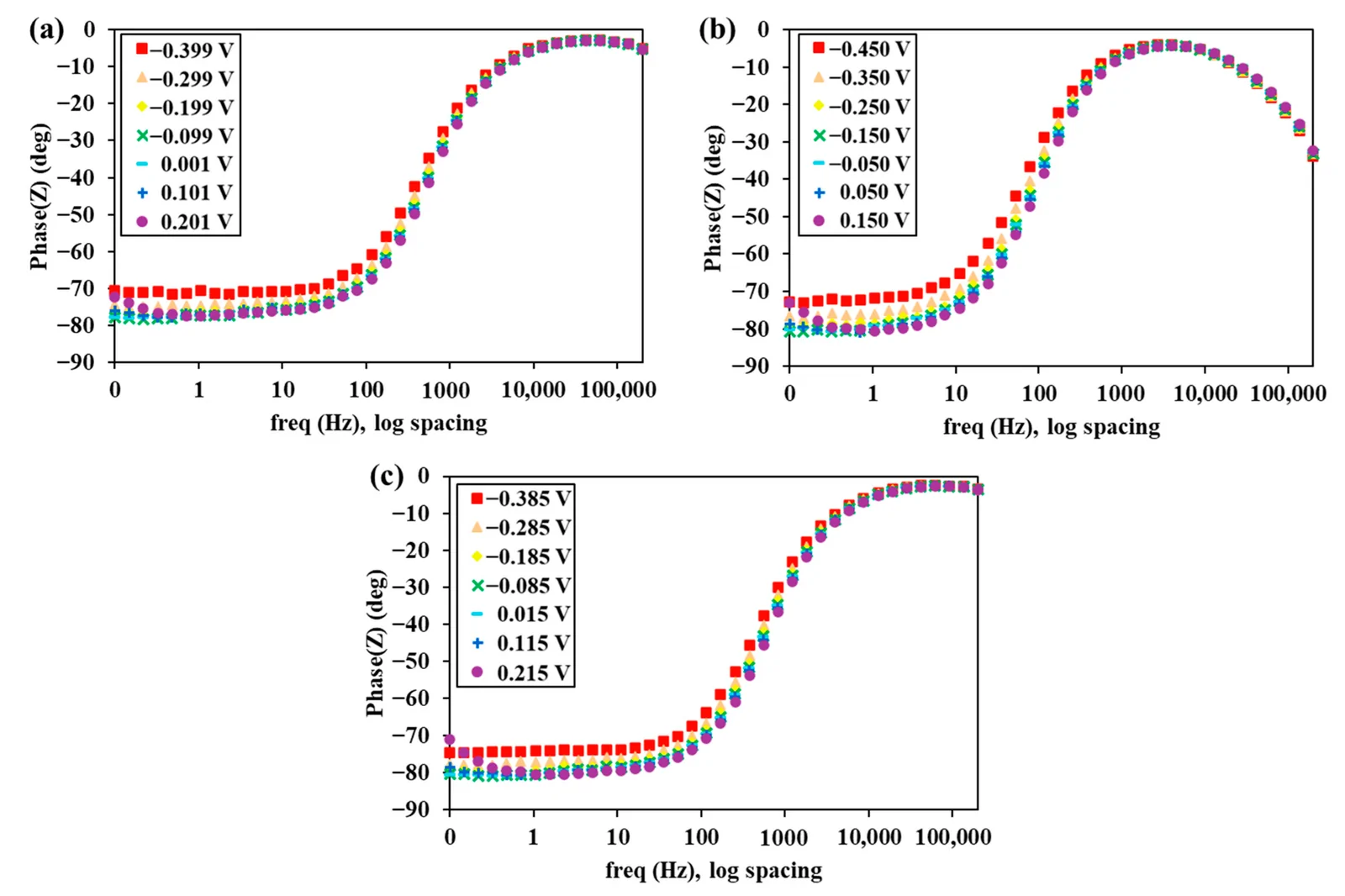
Bode-phase diagrams of experimental data for (**a**) VAR1, (**b**) VAR2 and (**c**) VAR3.

Figure 11 presents Bode-phase diagrams for samples VAR1, VAR 2 and VAR 3 at ±300 mV against the SCE. In all three samples, from high to low frequencies, the phase angle changes from values close to −5° for VAR1 and VAR2 and −40° for VAR3 to progressively more negative values. This change indicates that the system’s response is no longer dominated by the resistive contribution of the electrolyte and is now controlled by the metal-electrolyte film interface. As the applied potential becomes less negative in the medium-low frequency range, the phase angle values tend to grow more negative, reaching approximately −81° for VAR2 and VAR3 and −77° for VAR2. Additionally, the phase-angle curve shows that the electrochemical process takes place through a single time constant.

**Figure 12:**
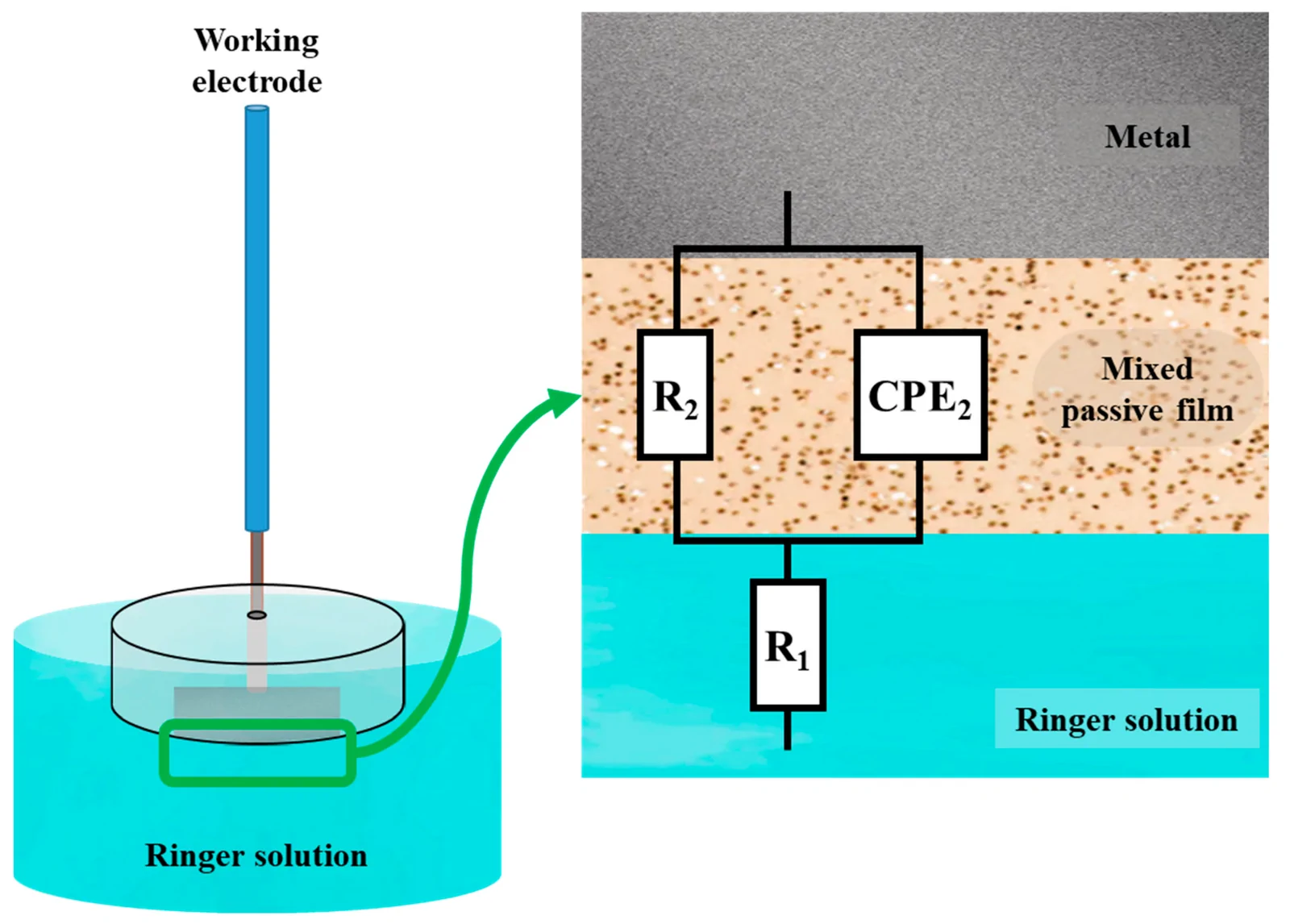
Electrical Equivalent circuit R_1_(CPE_2_R_2_) applied.

The applied equivalent electrical circuit model R_1_(CPE_2_ R_2_), which most closely matches the observed impedance data is displayed in Figure 12 and in Table 3. This model was selected because the Bode phase diagrams indicated the presence of only one time constant over the investigated frequency range. The resistance of the electrolyte, Ringer Solution (R_1_), the constant phase element (CPE_2_) and the resistance of the passive film (R_2_) were all represented by the model and the subsequent equation. Depending on the applied frequency (f), the CPE_2_, in this instance, can mimic resistance (n_2_ = 0), semi-infinite impedance of Warburg (n_2_ = 0.5) or a capacitor (n_2_ = 1) [52].

(1) $$Z\left(f\right)=R_{1}+\frac{R_{2}}{1+R_{2}{CPE}_{2}^{0}{\left(jw\right)}^{n_{2}}}$$

**Figure 13:**
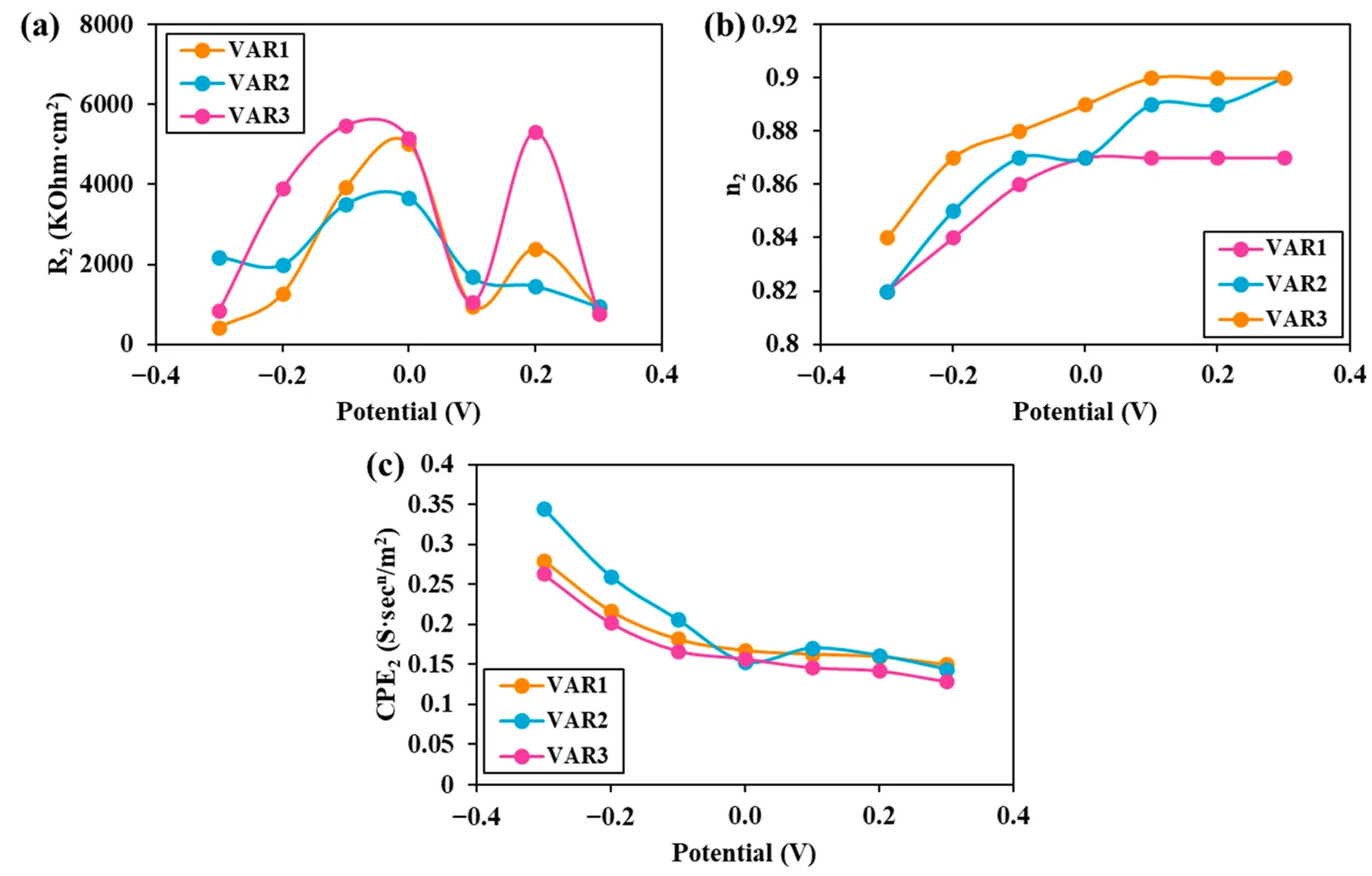
Evolution of (**a**) R_2_, (**b**) n_2_ and (**c**) CPE_2_ with the different potentials applied to the mixed passive film.

As illustrated in Figure 13, the application of these seven different potentials influences the values of various resistances and the constant phase element of the protective layer within the context of an electrical circuit. In this instance, the measured values align closely with the adjusted values, as evidenced by chi-squared values of approximately 10^−3^. It is possible to detect the existence of a mixed passive film structure by fitting the EIS spectra.

In all three samples, the R_2_ values increased to 0 V vs. SCE. Then, the R_2_ values increased and then decreased again to 0.3 V vs. SCE. The highest resistance values (R_p_ = R_1_ + R_2_) were observed for VAR3, followed by VAR2. This finding implies that adding Ta can increase the sample’s passive layer’s resistance to corrosion. As the concentration of Ta rises, the adjusted values of CPE_2_ decrease, suggesting that the formation of Ta_2_O_5_ may improve the compaction and the passive protective layer’s stability [53].

While not directly altering the substrate’s dendritic morphology (which results from the solidification process), Ta plays a key role in the chemical and electronic properties of the surface layer formed in the electrolyte. The more stable and compact passive film formed on Ta alloys (VAR3) changes the nature of the electrode/electrolyte interface. This manifests in the EIS data as a more pronounced capacitive behavior, indicated by the phase angles reaching closer to −90° (e.g., −81° for VAR3) and the formation of wider arcs in the Nyquist plots. The constant phase element behavior, which accounts for non-ideal capacitive responses, is influenced by the composition and homogeneity of this passive film, which is directly modulated by the Ta content.

The improved corrosion resistance with higher Ta content results from its influence on the passive film because it is highly reactive with oxygen and its presence in the alloy promotes the formation of a mixed oxide film on the surface. This film is composed of a complex mixture of oxides like TiO_2_, ZrO_2_, MoO_3_ and Ta_2_O_5_. The EIS results support this, as the CPE_2_ values (which relate to the capacitance/roughness of the film) decrease with increasing Ta concentration, suggesting a more compact and uniform layer. This denser oxide layer acts as a superior barrier to the transport of aggressive ions (like Cl^−^ from the Ringer’s solution) towards the metal surface, thus hindering the electrochemical dissolution process. This mechanism is evidenced by the significantly lower corrosion current density (i_corr_) and corrosion rate (CR) for VAR3 compared to VAR1 and VAR2. The potentiodynamic curves show lower anodic current densities for VAR3, and the EIS data shows higher polarization resistance (R_p_), which is a direct measure of the film’s protective ability.

## 4. Conclusions

In this work, the influence of Ta content on the microstructure, microhardness and electrochemical behavior of Ti–Mo–Zr–xTa alloys obtained through Vacuum Arc Remelting (VAR) was studied:

- Through the VAR process, the production of alloys with an overall redistribution of the constituent elements was desired. However, the EDS elemental maps did not reveal pronounced large-scale elemental accumulation at the spatial scale investigated; therefore fine-scale segregation between dendritic and interdendritic regions cannot be excluded. The patterns of X-ray diffraction revealed that β was the primary phase in every composition. Further, when the Ta content increased from 5% to 15%, the stability of this phase increased and the amount of the martensitic α″ phase decreased.
- The microhardness tests showed that Ta content decreased the microhardness, with the VAR1 sample showing the greatest values. Reduced load measurements were shown to provide better reproducibility of results and improved resistance to local surface inhomogeneities.
- In Ringer solution, all three compositions presented the development of a stable passive film after 24 h of immersion, demonstrating good resistance to the more aggressive environment. VAR3 showed the lowest corrosion rate as well as the highest values of polarization resistance. This behavior is possibly related to the influence of Ta on the passive film, making it more compact and resistant and promoting the development of protective oxides.

According to the results obtained, the Ti–Mo–Zr–15Ta alloy presented the best electrochemical behavior, showing the lowest corrosion rate and the highest polarization resistance, while the Ti–Mo–Zr–5Ta alloy showed the highest microhardness values. Therefore, the alloy with 15% Ta may be more suitable for orthopedic and dental implant components that are exposed for long periods to physiological fluids, where corrosion resistance is an important requirement. The absence of Al and V and the good electrochemical stability of these alloys provide a good basis for the development of new metallic biomaterials, although further mechanical, wear and biological tests are required.

## Figures and Tables

**Table 1:** Research samples’ microhardness values and footprint depth after applying 5 gf, 25 gf and 50 gf.

Samples	Load (gf)	Microhardness (HV)					Depth (μm)	CV (%)
		Mean	Median	SD	Maximum	Minimum		
VAR1	5	336	335	15	360	309	1.06	4.41
	25	323	327	11	343	306	2.42	3.50
	50	313	311	9	326	297	3.48	3.03
VAR2	5	331	327	18	361	310	1.07	5.57
	25	323	322	10	344	311	2.42	3.03
	50	311	310	8	326	300	3.49	2.55
VAR3	5	325	328	20	350	290	1.08	6.16
	25	308	308	8	321	294	2.48	2.60
	50	301	301	10	315	285	3.54	3.22

**Table 2:** Corrosion results of TiMoZrxTa samples studied after applying Tafel Fit.

Corrosion Parameters	VAR1	VAR2	VAR3
Equivalent weight (g/eq)	13.63	13.88	14.44
Density (g/cm^3^)	6.11	6.71	7.32
Area (cm^2^)	1.38	1.46	0.96
E_corr_ (V)	−0.26	−0.34	−0.22
i_corr_ (µA/cm^2^)	1.45 × 10^−2^	1.58 × 10^−2^	3.13 × 10^−3^
β_c_ (V)	0.13	0.07	0.09
β_a_ (V)	0.41	0.61	0.12
CR (mm/year)	1.05 × 10^−4^	1.07 × 10^−4^	4.82 × 10^−6^

**Table 3:** Parameters obtained after applying the R1(CPE2R2) equivalent circuit with different potentials for the mixed passive film.

Parameters	Samples	−0.3 V	−0.2 V	−0.1 V	0 V	0.1 V	0.2 V	0.3 V
R_2_ (KOhm·cm^2^)	VAR1	423	1268	3929	5012	959	2390	908
	VAR2	2181	2000	3500	3658	1684	1461	948
	VAR3	840	3901	5487	5155	1048	5312	762
n_2_	VAR1	0.82	0.84	0.86	0.87	0.87	0.87	0.87
	VAR2	0.82	0.85	0.87	0.87	0.89	0.89	0.9
	VAR3	0.84	0.87	0.88	0.89	0.9	0.9	0.9
Y_2_ (S·sec^n^/cm^2^)	VAR1	2.80 × 10^−5^	2.17 × 10^−5^	1.82 × 10^−5^	1.68 × 10^−5^	1.63 × 10^−5^	1.60 × 10^−5^	1.50 × 10^−5^
	VAR2	3.45 × 10^−5^	2.60 × 10^−5^	2.06 × 10^−5^	1.53 × 10^−5^	1.70 × 10^−5^	1.61 × 10^−5^	1.44 × 10^−5^
	VAR3	2.63 × 10^−5^	2.02 × 10^−5^	1.67 × 10^−5^	1.57 × 10^−5^	1.46 × 10^−5^	1.42 × 10^−5^	1.29 × 10^−5^

## Data Availability

The original contributions presented in this study are included in the article. Further inquiries can be directed to the corresponding author.
